# Costs of Public Pharmaceutical Services in Rio de Janeiro Compared to *Farmácia Popular* Program

**DOI:** 10.1590/S1518-8787.2016050006605

**Published:** 2016-11-24

**Authors:** Rondineli Mendes da Silva, Rosângela Caetano

**Affiliations:** I Departamento de Política de Medicamentos e Assistência Farmacêutica. Escola Nacional de Saúde Pública Sérgio Arouca. Fundação Oswaldo Cruz. Rio de Janeiro, RJ, Brasil; II Departamento de Planejamento e Administração em Saúde. Instituto de Medicina Social. Universidade do Estado do Rio de Janeiro. Rio de Janeiro, RJ, Brasil

**Keywords:** Pharmaceutical Services, economics, Drug Costs, Drugs, Essential, supply & distribution, Costs and Cost Analysis, Health Economics, National Policy of Pharmaceutical Assistance

## Abstract

**OBJECTIVE:**

To analyze the costs of public pharmaceutical services compared to *Farmácia Popular* Program (Popular Pharmacy Program).

**METHODS:**

Comparison between prices paid by *Aqui Tem Farmácia Popular* Program (Farmácia Popular is available here) with the full costs of medicine provision by the Municipal Health Department of Rio de Janeiro. The comparison comprised 25 medicines supplied by both the municipal pharmaceutical service and *Aqui Tem Farmácia Popular* Program. Calculating the cost per pharmaceutical unit of each medicine included expenditure by Municipal Health Department of Rio de Janeiro with procurement (price), logistics, and local dispensation. The reference price of medicines paid by *Aqui Tem Farmácia Popular* was taken from the Brazilian Ministry of Health standard in force in 2012. Comparisons included full reference price; reference price minus 10.0% copayment by users; and maximum reference paid by the Ministry of Health (minus copayment and taxes). Simulations were carried out of the differences between the costs of Municipal Health Department of Rio de Janeiro with the common medicines and those potentially incurred based on the reference price of *Aqui Tem Farmácia Popular*.

**RESULTS:**

The Municipal Health Department of Rio de Janeiro spent R$28,526,526.57 with 25 medicines of the common list in 2012; 58.7% accounted for direct procurement costs. The estimated costs of the Health Department were generally lower than the reference prices of the *Aqui Tem Farmácia Popular* Program for 20 medicines, regardless of reference prices. The potential costs incurred by Health Department if expenditure of its consumption pattern were based on the reference prices of *Aqui Tem Farmácia Popular* would be R$124,170,777.76, considering the best scenario of payment by the Brazilian Ministry of Health (90.0% of the reference price, minus taxes).

**CONCLUSIONS:**

The difference in costs between public provision by Municipal Health Department of Rio de Janeiro and *Farmácia Popular* Program indicates that some reference prices could be reviewed aiming at their reduction.

## RESUMO

**OBJETIVO:**

Analisar custos da assistência farmacêutica pública frente ao Programa Farmácia Popular.

**MÉTODOS:**

Comparação entre os valores pagos pelo Programa Aqui Tem Farmácia Popular com os custos integrais relativos à provisão de medicamentos pela Secretaria Municipal de Saúde do Rio de Janeiro. A comparação compreendeu 25 medicamentos, comuns tanto à provisão pela assistência farmacêutica pública municipal quanto pelo Programa Aqui Tem Farmácia Popular. O cálculo do custo unitário por unidade farmacotécnica de cada medicamento envolveu os gastos da Secretaria Municipal de Saúde com custos de aquisição (preço), logísticos e com a dispensação em nível local. O valor de referência dos medicamentos pago pelo Aqui Tem Farmácia Popular foi extraído da norma ministerial em vigor em 2012. As comparações envolveram o valor de referência pleno; valor de referência com desconto dos 10,0% pagos de contrapartida pelos usuários; e valor de referência máximo pago pelo Ministério da Saúde (descontados contrapartida e sem impostos).Foram realizadas simulações das diferenças entre os gastos da Secretaria Municipal de Saúde do Rio de Janeiro com os medicamentos do elenco comum e os que seriam incorridos se esses tivessem sido executados com base no valor de referência do Aqui Tem Farmácia Popular.

**RESULTADOS:**

A Secretaria Municipal de Saúde do Rio de Janeiro gastou R$28.526.526,57 com 25 medicamentos do rol comum em 2012; 58,7% corresponderam a custos diretos com a aquisição dos produtos. Os custos estimados da Secretaria Municipal de Saúde do Rio de Janeiro foram, em geral, menores que os valores de referência do Programa Aqui Tem Farmácia Popular em 20 medicamentos, independentemente dos valores de referência. Os custos que seriam incorridos pela Secretaria Municipal de Saúde do Rio de Janeiro, caso seu padrão de consumo tivesse como valor de pagamento os valores de referência do Aqui Tem Farmácia Popular seriam de R$124.170.777,76 considerando a melhor situação de pagamento pelo Ministério da Saúde (90,0% do valor de referência, com impostos descontados).

**CONCLUSÕES:**

A diferença de custos entre a provisão pública pela Secretaria Municipal de Saúde do Rio de Janeiro e o Programa Aqui Tem Farmácia Popular sinaliza que alguns valores de referência poderiam ser objetos de exame para sua redução.

## INTRODUCTION

Difficulties in access to medicines due to their high share in private household spending in Brazil^16^ and supply problems in public units of the Brazilian Unified Health System (SUS)^2^ justified the introduction of *Farmácia Popular* Program (PFPB) by the Brazilian Ministry of Health (MH) in 2004^13^. Its creation, however, did not alter the responsibilities of municipalities in the provision of medicines from SUS.

The program expanded into arrangements involving the public and private sectors. It has its own network of pharmacies and a public-private partnership with the retail pharmaceutical sector, both of them with or without copayment by users.

The number of pharmacies accredited by the private division of PFPB, called *Aqui Tem Farmácia Popular*, increased over 750% in 2006-2013 and was responsible for the program’s geographical spread^15^. It has its own rules of operation and includes 41 products to treat the most prevalent diseases in the population, most of which feature in the basic list provided by pharmacies from the SUS network. A list of antihypertensive, antidiabetic and antiasthmatic medicines is exempt from co-financing^3^ ^,^ ^13^ ^,^ ^15^.

The Ministry of Health’s direct disbursement to the accredited retail network is based on a reference price (RP) per medicine. This price is valid for the entire country and comprises, in addition to the procurement price, costs related to logistics, dispensation and maintenance of pharmacies, insurance and taxes.

The costs of the ATFP Program are subject to questioning within the public model for provision of access. An audit by *Tribunal de Contas da União* (TCU – Federal Court of Accounts) raised the debate on the differences in prices found in public tenders compared to those in the Program. The audit identified a huge discrepancy between the RP paid by ATFP and the prices of 13 medicines purchased by the public sector in April 2010. For four of them, the difference between the RP and the average procurement price exceeded 1,000%^a^. TCU pointed out that simple comparison with bidding prices was not enough to establish whether one program is more efficient than the other, and therefore studies considering other costs involved are necessary.

New medicine supply arrangements in Brazil are important strategies to back policies aimed at expanding access. However, they lack data to support analyses of funding sustainability and more efficient use of public resources.

This study aimed to analyze the costs of public pharmaceutical services compared to *Farmácia Popular* Program.

## METHODS

A comparative study of costs (in reais – R$) of the two models of pharmaceutical services (PS), the ATFP Program and public services, in Rio de Janeiro, Southeastern Brazil, in 2012.

*Secretaria Municipal de Saúde do Rio de Janeiro* (SMS-RJ – Municipal Health Department of Rio de Janeiro) has a recognized track record of efficiency in the procurement of medicines^b^ and was one of the first in Brazil to make purchases using the price registration system^8^ ^,^ ^20^. In 2012, it had a network of 270 health units of varying complexity, 201 of primary health care^10^ ^,^ ^c^. Coverage by *Estratégia de Saúde da Família* (Family Health Strategy) reached around 40.0%, marked by recent expansion arising from the creation of new units (*Clínicas da Família* – Family Clinics) managed by social organizations (SO) and including pharmacies within their structure^7^.

The comparative analysis involved 25 medicines common to public municipal pharmaceutical services and the ATFP Program, according to the lists contained in Ordinances 1555/2013^d^, which funds the *Componente Básico da Assistência Farmacêutica* (CBAF – Basic Component of Pharmaceutical Services), and 971/2012^e^, related to *Aqui Tem Farmácia Popular*.

Calculating the cost per pharmaceutical unit (PU) of each medicine included SMS-RJ costs with procurement, logistics (storage, distribution and transport) and local dispensation. Administrative costs involved in procurement or loss and misplacement were not computed.

Procurement costs were obtained from the price registration minutes of SMS-RJ bidding processes, published in the Municipal Official Gazette, following the methodology used by Silva and Caetano^14^. The publication features the unit price for each item.

The source of logistics costs was the SMS-RJ outsourcing contract for this activity. The amount paid to the company was prorated to all medicines, considering the following specific adjustments: (i) monthly consumption of each medicine in PU; (ii) storage and transport space (in m^3^ stored and shipped); (iii) labor force employed in separating medicines, considering the number of items per order; and (iv) insurance, considering the average value in stock. This allowed isolating the contribution of each medicine of the common list between municipal public provision and the ATFP Program, enabling the individualization of a price per PU linked to logistics costs.

Dispensation costs involved expenses related to human resources of basic network pharmaceutical services; purchase of materials, furniture and equipment; and building management and maintenance services. The result of this cost component was subsequently prorated to isolate the costs with the PU of each medicine.

Staff costs were based on a specific SMS-RJ census of September 2012, which identified the number of personnel dedicated to pharmacy, separated by professional category – pharmaceutical and support staff (pharmacy technician, administrative personnel, among others) – and employment relationship (direct municipal administration and SO). Civil servant salaries were determined from the municipal administration salary scale related to the average monthly remuneration values of the corresponding levels, including hazard pay, three-year service bonus and transportation allowance. The salaries of professionals hired by SO were obtained from the SMS-RJ internal system (*Painel de Gestão das Parcerias com Organizações Sociais* – OS INFO system [Social Organization Partnership Management Panel]), used to monitor and evaluate the management contracts of those organizations. The computation included wages and benefit values and other labor costs such as provision, Government Severance Indemnity Fund for Employees, *Instituto Nacional do Seguro Social* (National Social Security Institute), *Programa de Integração Social* (Social Integration Program) and *Programa de Formação do Patrimônio do Servidor* (Public Servant Savings Program).

Data from OS INFO system were used to estimate costs with equipment/furniture (air conditioning; refrigerators; computers and printers; bookcases; tables and chairs, among others), material (paper, pens, prescription pads, etc.), building management expenses and other administrative costs. The SMS-RJ Family Clinics follow a standard structure and operation model. Amounts and values estimated for a FC were arbitrarily extrapolated to 201 basic units. Furniture and equipment costs were based on the lowest unit purchase price recorded in the 2012 OS INFO system and depreciated according to the Regulatory Instructions of the Brazilian Internal Revenue Service^f^ ^,^ ^g^.

An apportionment method was applied to the administrative costs of service contracts and maintenance, which cannot be individually measured by service item. Physical area (m^2^) was used for energy, water and sewage, cleaning/maintenance, security and building maintenance; quantity of equipment for air conditioning and refrigeration; and number of network access points in pharmacies and number of logins to access Internet service providers and electronic medical record systems, respectively.

Dispensation costs per PU were estimated according to the formula below. The percentage share of each product in the common list was calculated from the total expenditure of SMS-RJ with the procurement of basic medicines in 2012. This percentage share was applied to the total dispensation cost and weighted by the annual consumption of each medicine, obtained from the municipal Medicine Distribution Center.

Where


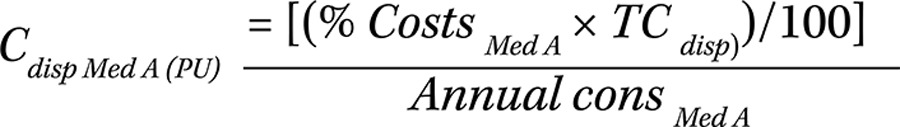


C_*disp Med A (PU)*_ *=* cost of medicine A in dispensation stage

TC_*disp*_ = Total dispensation costs

Annual cons_*Med A*_ = Annual consumption of medicine A in 2012

Estimate of the final cost per PU to each medicine of the SMS-RJ corresponded to the sum of the cost components of procurement, logistics and dispensation for each one of the 25 medicines.

The RP of medicines in the common list paid by ATFP was obtained from Ordinance 971, in force in 2012^e^.

Comparisons between the RP and the estimated cost per PU of SMS-RJ were based on the percentage variation between both of them. Three situations were considered: full RP, disregarding copayment percentages; RP minus 10.0% copayment by users; and maximum RP paid by MH minus taxes, that is, minus 4.27% taxes on the gross revenue of private pharmacies in 2011^h^.

Based on the annual consumption of SMS-RJ, simulations were performed on the differences between SMS costs with each medication of the common list and those potentially incurred based on the RP of ATFP.

Excel^©^ software was used to estimate costs and comparisons.

The study was approved by the Ethics Committees of the Institute of Social Medicine – Rio de Janeiro State University (Opinion 170,617/2012) and of SMS-RJ (Opinion 350A/2012).

## RESULTS

SMS-RJ spent R$28,526,526.57 on medications of the common list in 2012, 58.7% of which with the direct procurement of products (Table 1). The average total cost per PU was R$ 1.6386, ranging from R$0.0173 to R$12.3647. Five medicines accounted for 51.4% of expenditure: metformin (850 mg); NPH human insulin; enalapril; losartan; beclomethasone (250 mcg).

**Table 1:** Estimated costs with procurement, logistics and dispensation of Municipal Health Department per pharmaceutical unit of medicines in the common list of the ATFP Program, total and by cost component (in R$). Rio de Janeiro, RJ, Southeastern Brazil, 2012.

Common list medicines	PU Consumption 2012	PU Procurement price	Procurement costs 2012	PU logistics cost	Logistics costs 2012	PU dispensation costs	Dispensation costs 2012	Total PU cost	Total SMS-RJ cost 2012
Alendronate sodium 70 mg pill	290,040	0.2698	78,252.79	0.1620	46,986.48	0.1045	30,309.18	0.5363	155,548.45
Atenolol 25 mg pill	40,146,204	0.0117	469,710.59	0.0087	349,271.97	0.0108	433,579.00	0.0312	1,252,561.56
Ipratropium Bromide 0.02 mg/dose	1,219,200	0.0776	94,609.92	0.0175	21,336.00	0.1173	143,012.16	0.2124	258,958.08
Ipratropium Bromide 0.25 mg/ml	1,789,440	0.0272	48,672.77	0.0105	18,789.12	0.0111	19,862.78	0.0488	87,324.67
Budesonide 50 mcg/nasal spray dose	18,000,000	0.0820	1,476,000.00	0.0047	84,600.00	0.0230	414,000.00	0.1097	1,974,600.00
Captopril 25 mg pill	78,002,640	0.0102	795,626.93	0.0050	390,013.20	0.0036	280,809.50	0.0188	1,466,449.63
Carbidopa 25 mg+Levodopa 250 mg pill	253,440	0.1598	40,499.71	0.0300	7,603.20	0.2485	62,979.84	0.4383	111,082.75
Benserazide hydrochloride 25 mg +Levodopa 100 mg pill	240,000	1.0500	252,000.00	0.0600	14,400.00	0.3761	90,264.00	1.4861	356,664.00
Metformin hydrochloride 500 mg pill	7,440,000	0.0429	319,176.00	0.0300	223,200.00	0.0482	358,608.00	0.1211	900,984.00
Metformin hydrochloride 850 mg pill	52,466,280	0.0349	1,831,073.17	0.0200	1,049,325.60	0.0200	1,049,325.60	0.0749	3,929,724.37
Propranolol hydrochloride 40 mg pill	3,870,480	0.0097	37,543.66	0.0200	77,409.60	0.0068	26,319.26	0.0365	141,272.52
Beclomethasone dipropionate 250 mcg/spray dose	12,720,000	0.1200	1,526,400.00	0.0080	101,760.00	0.0343	436,296.00	0.1623	2,064,456.00
Beclomethasone dipropionate 50 mcg/spray dose	4,800,000	0.1400	672,000.00	0.0163	78,240.00	0.0658	315,840,00	0.2221	1,066,080.00
Ethinyl Estradiol 0.15 mg + Levonorgestrel 0.03 mg pill	131,604	0.4500	59,221.80	1.2900	169,769.16	0.0429	5,645.81	1.7829	234,636.77
Glibenclamide 5 mg pill	43,710,216	0.0070	305,971.51	0.0100	437,102.16	0.0022	96,162.48	0.0192	839,236.15
Hydrochlorothiazide 25 mg pill	68,826,204	0.0094	646,966.32	0.0040	275,304.82	0.0039	268,422.20	0.0173	1,190,693.33
NPH Human Insulin 100 UI/ml vial 10 ml	324,000	8.7800	2,844,720.00	0.3600	116,640.00	2.4004	777,729.60	11.5404	3,739,089.60
Regular Human Insulin 100 UI/ml vial 10 ml	86,400	9.3500	807,840.00	0.9900	85,536.00	2.0247	174,934.08	12.3647	1,068,310.08
Losartan potassium 50 mg pill	42,000,000	0.0330	1,386,000.00	0.0100	420,000.00	0.0082	344,400.00	0.0512	2,150,400.00
Enalapril Maleate 10 mg pill	104,937,840	0.0145	1,521,598.68	0.0014	146,912.98	0.0107	1,122,83.89	0.0266	2,791,346.54
Timolol Maleate 5 mg/ml (0.5%)	92,760	0.1940	17,995.44	0.2020	18,737.52	0.0851	7,893.88	0.4811	44,626.84
Norethisterone 0.35 mg pill	72,348	4.5150	326,651.22	0.0300	2,170.44	0.3074	22,239.78	4.8524	351,061.44
Simvastatin 20 mg pill	10,800,000	0.0389	420,120.00	0.0600	648,000.00	0.0276	298,080.00	0.1265	1,366,200.00
Sulfate Salbutamol 100 mcg/spray dose	9,636,000	0.0149	143,576.40	0.0058	55,888.80	0.0090	86,724.00	0.0297	286,189.20
Estradiol 50 mg/ml + Norethisterone 5 mg/ml amp	113,196	5.1500	582,959.40	0.4900	55,466.04	0.5354	60,605.14	6.1754	699,030.58

Total	501,968,292	-	16,705,186.30	-	4,894,463.09	-	6,926,877.18	-	28,526,526.57

Amp: ampoule; ATPF: *Aqui Tem Farmácia Popular*; SMS-RJ: *Secretaria Municipal de Saúde do Rio de Janeiro* (Municipal Health Department of Rio de Janeiro); PU: pharmaceutical unit

There was significant variation in the percentage share of cost components, especially for some specific products (Figure). For most medicines, procurement costs were the highest, accounting for more than 70.0% of costs in the following products: norethindrone; estradiol + norethisterone; NPH and regular human insulin; beclomethasone (250 mcg); budesonide and benserazide + levodopa.

**Figure:**
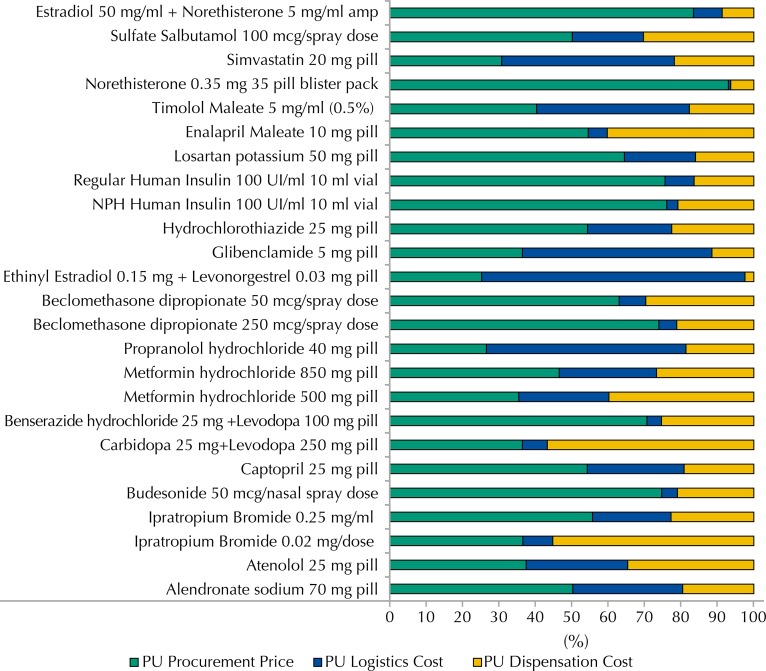
Contribution of cost components (in R$) to the total SMS-RJ price per pharmaceutical unit of the common list medicines of the ATFP Program. Rio de Janeiro, RJ, Southeastern Brazil, 2012.

Amp: ampoule; ATPF: *Aqui Tem Farmácia Popular*; SMS-RJ: *Secretaria Municipal de Saúde do Rio de Janeiro* (Municipal Health Department of Rio de Janeiro); PU: pharmaceutical unit

Logistics costs predominated in three products: ethinylestradiol + levonorgestrel (72.4%); propranolol (54.8%), and glibenclamide (52.1%). Ipratropium spray and carbidopa + levodopa showed proportionally higher dispensation costs than the others.

Comparing the costs of SMS-RJ and the RP of ATFP Program, municipal costs were generally lower, regardless of the RP considered (Table 2). The difference between full RP and cost per PU in SMS-RJ was 279.8% higher. With the 10% copayment discount, variations were slightly lower on average: 270.7%. In the most favorable comparison with ATFP (minus copayment and taxes), the percentage difference was 3.5 times lower than the federal program, on average.

**Table 2:** Comparison between the costs of Municipal Health Department and the reference price per pharmaceutical unit of medicines in the common list of the ATFP Program. Rio de Janeiro, Southeastern Brazil, 2012.

Common list medicines	RP	Max. RP paid MH	RP minus taxes	% dif. between full RP and SMS Cost	% dif. between max. RP paid by MH and SMS Cost	% dif. between max. RP paid by MH minus taxes and SMS Cost
Alendronate sodium 70 mg pill	3.7400	3.3700	3.2261	597.4	528.4	501.5
Atenolol 25 mg pill	0.1900	0.1900	0.1819	509.0	509.0	483.0
Ipratropium Bromide 0.02 mg/dose	0.0600	0.0600	0.0574	-71.8	-71.8	-73.0
Ipratropium Bromide 0.25 mg/ml	0.2700	0.2700	0.2585	453.3	453.3	429.7
Budesonide 50 mcg/nasal spray dose	0.1300	0.1200	0.1149	18.5	9.4	4.7
Captopril 25 mg pill	0.2800	0.2800	0.2680	1,389.4	1,389.4	1.325.8
Carbidopa 25 mg+Levodopa 250 mg pill	0.6400	0.5800	0.5552	46.0	32.3	26.7
Benserazide hydrochloride 25 mg +Levodopa 100 mg pill	1.1700	1.0500	1.0052	-21.3	-29.3	-32.4
Metformin hydrochloride 500 mg pill	0.1300	0.1300	0.1244	7.3	7.3	2.8
Metformin hydrochloride 850 mg pill	0.1600	0.1600	0.1532	113.6	113.6	104.5
Propranolol hydrochloride 40 mg pill	0.0800	0.0800	0.0766	119.2	119.2	109.8
Beclomethasone dipropionate 250 mcg/spray dose	0.1500	0.1500	0.1436	-7.6	-7.6	-11.5
Beclomethasone dipropionate 50 mcg/spray dose	0.1300	0.1300	0.1244	-41.5	-41.5	-44.0
Ethinyl Estradiol 0.15 mg + Levonorgestrel 0.03 mg pill	4.1900	3.7700	3.6090	135.0	111.5	102.4
Glibenclamide 5 mg pill	0.1200	0.1200	0.1149	525.0	525.0	498.3
Hydrochlorothiazide 25 mg pill	0.0800	0.0800	0.0766	362.4	362.4	342.7
NPH Human Insulin 100 UI/ml 10 ml vial	26.5500	26.5500	25.4163	130.1	130.1	120.2
Regular Human Insulin 100 UI/ml 10 ml vial	26.5500	26.5500	25.4163	114.7	114.7	105.6
Losartan potassium 50 mg pill	0.3200	0.3200	0.3063	525.0	525.0	498.3
Enalapril Maleate 10 mg pill	0.3900	0.3900	0.3733	1,366.2	1,366.2	1,303.6
Timolol Maleate 5 mg/ml (0.5%)	0.9600	0.8600	0.8233	99.5	78.8	71.1
Norethisterone 0.35 mg pill	4.9600	3.7700	3.6090	2.2	-22.3	-25.6
Simvastatin 20 mg pill	0.5100	0.4600	0.4404	303.2	263.6	248.1
Sulfate Salbutamol 100 mcg/spray dose	0.1000	0.1000	0.0957	236.7	236.7	222.3
Estradiol 50 mg/ml + Norethisterone 5 mg/ml amp	11.3100	10.1700	9.7357	83.1	64.7	57.7
Average difference				279.8	270.7	254.9

Amp: ampoule; ATPF: *Aqui Tem Farmácia Popular*; Dif.: difference; MH: Brazilian Ministry of Health; SMS-RJ: *Secretaria Municipal de Saúde do Rio de Janeiro* (Municipal Health Department of Rio de Janeiro); RP: reference price; PU: pharmaceutical unit

Captopril and enalapril showed percentage differences above 1,000%, with a 14 times lower cost in the municipal context, regardless of the comparison scenario with ATFP. Five other medicines also stood out favorably in the municipal dispensation, with costs at least five times lower than ATFP, even after discounting copayment and taxes: alendronate sodium, losartan, glibenclamide, atenolol and ipratropium solution (Table 2).

Simulations of potential costs incurred by SMS-RJ if its medicine consumption pattern were paid according to the RP of ATFP showed values of R$124,170,777.76, considering the best payment scenario by MH (90,0% of RP minus taxes). SMS-RJ would spend 3.4 times more than estimated with the entire common list if it used the RP of ATFP (Table 3).

**Table 3:** Total cost (in R$) of Municipal Health Department based on estimated costs per pharmaceutical unit and simulation of expenses considering the reference prices of the ATFP Program, for medicines in the common list. Rio de Janeiro, RJ, Southeastern Brazil, 2012.

Common list medicines	SMS-RJ annual consumption	SMS-RJ total estimated cost	SMS-RJ total expenses simulated by ATFP full RP^a^	SMS-RJ total expenses simulated by ATFP^b^ max RP paid by MH^b^	SMS-RJ total expenses simulated by max. RP minus taxes^c^
Alendronate sodium 70 mg pill	290,040	155,548.45	1,084,749.60	977,434.80	935.698.33
Atenolol 25 mg pill	40,146,204	1,252,561.56	7,627,778.76	7,627,778.76	7.302.072.61
Ipratropium Bromide 0.02 mg/dose	1,219,200	258,958.08	73,152.00	73,152,00	70.028.41
Ipratropium Bromide 0.25 mg/ml	1,789,440	87,324.67	483,14.,80	483,148.80	462.518.35
Budesonide 50 mcg/nasal spray dose	18,000,000	1,974,600.00	2,340,000.00	2,160,000.00	2.067.768.00
Captopril 25 mg pill	78,002,640	1,466,449.63	21,840,739.20	21,840,739.20	20.908.139.64
Carbidopa 25 mg+Levodopa 250 mg pill	253,440	111,082.75	162,201.60	146,995.20	140.718.50
Benserazide hydrochloride 25 mg + Levodopa 100 mg pill	240,000	356,664.00	280,800.00	252,000,00	241.239.60
Metformin hydrochloride 500 mg pill	7,440,000	900,984.00	967,200.00	967,200.00	925.900.56
Metformin hydrochloride 850 mg pill	52,466,280	3,929,724.37	8,394,60.,80	8,394,604.80	8.036.155.18
Propranolol hydrochloride 40 mg pill	3,870,480	141,272.52	309,638.40	309,638.40	296.416,84
Beclomethasone dipropionate 250 mcg/spray dose	12,720,000	2,064,456.00	1,908,000.00	1,908,000.00	1.826.528.40
Beclomethasone dipropionate 50 mcg/spray dose	4,800,000	1,066,080.00	624,000.00	624,000.00	597.355.20
Ethinyl Estradiol 0.15 mg + Levonorgestrel 0.03 mg pill	131,604	234,636,77	551,420.76	496,147.08	474.961.60
Glibenclamide 5 mg pill	43,710,216	839,236.15	5,245,225.92	5,245,225.92	5.021.254.77
Hydrochlorothiazide 25 mg pill	68,826,204	1,190,693.33	5,506,096.32	5,506,096.32	5.270.986.01
NPH Human Insulin 100 UI/ml 10 ml vial	324,000	3,739,089.60	8,602,200.00	8,602,200.00	8.234.886.06
Regular Human Insulin 100 UI/ml 10 ml vial	86,400	1,068,310,08	2,293,920.00	2,293,920.00	2.195.969,62
Losartan potassium 50 mg pill	42,000,000	2,150,400.00	13,440,000.00	13,440,000,00	12.866.112.00
Enalapril Maleate 10 mg pill	104,937,840	2,791,346.54	40,925,757.60	40,925,757.60	39.178.227.75
Timolol Maleate 5 mg/ml (0.5%)	92,760	44,626,84	89,049.60	79,773.60	76.367.27
Norethisterone 0.35 mg pill	72,348	351,061.44	358,846.08	272,751.96	261.105.45
Simvastatin 20 mg pill	10,800,000	1,366,200.00	5,508,000.00	4,968,000.00	4.755.866.40
Sulfate Salbutamol 100 mcg/spray dose	9,636,000	286,189.20	963,600.00	963,600.00	922.454.28
Estradiol 50 mg/ml + Norethisterone 5 mg/ml amp	113,196	699,030.58	1,280,246.76	1,151,203.32	1.102.046.94

Total		28,526,526.57	130,860,376.20	129,709,367.76	124,170,777.76

Amp: ampoule; ATPF: *Aqui Tem Farmácia Popular*; MH: Brazilian Ministry of Health; SMS-RJ: *Secretaria Municipal de Saúde do Rio de Janeiro* (Municipal Health Department of Rio de Janeiro); PU: pharmaceutical unit; RP: reference price

^a^ Simulation using full reference price paid by ATFP, disregarding copyament percentages.

^b^ Simulation using reference price minus 10% copayment by users.

^c^ Simulation using maximum reference price paid by MH minus taxes.

## DISCUSSION

Comparison between the reference price paid to establishments accredited by the Brazilian Ministry of Health in the ATFP Program and the costs of public supply of common list medications by SMS-RJ, calculated per pharmaceutical unit, in general showed differences in favor of the municipal public service, which were 3.5 times greater, on average. The cost of municipal provision was lower in 20 of the 25 items in common with the ATFP Program. Considering the demand of each drug consumed in 2012 by SMS-RJ, the simulation showed that if the municipality had acquired them for the lowest RP, it would incur over R$95 million in the overall cost for the same 25 products.

The ATFP Program offers medicines fully covered by CBAF for use in primary health care. Nationwide expenditure with ATFP totaled R$1,293,874,112.05 in 2012, of which approximately R$261 million were used exclusively to pay pharmacies established in Rio de Janeiro, RJ^i^. This volume of resources reinforces the importance of analyzing such expenditures vis-à-vis those of other agencies, especially municipal ones, which bear the heavier burden in providing pharmaceutical services related to primary care.

For most products, procurement costs were the highest compared to the other components investigated. A common feature of medicines with procurement costs above 70.0% of the total cost per PU was low volume purchases.

The contraceptives levonorgestrel and estradiol + norethisterone are acquired centrally by MH and passed on to the municipality, with low volume purchases by SMS. A similar condition occurs with insulin, which is only purchased by the municipality when the MH supply is disrupted. Beclomethasone and budesonide, indicated for treatment of asthma and rhinitis, have little market competition due to the low number of products and manufacturers in the country and the absence of generics, and were only incorporated into SMS-RJ procurement in 2011. Benserazide + levodopa had a single manufacturer until 2012, which classified it as a unique product, hindering price negotiations.

Low volume purchases and less bargaining power contribute to higher prices and inefficient medicine purchases in SUS^19^. Higher volume purchases attract greater interest from suppliers, expand competition, and are usually associated with price reductions. They tend to attract manufacturers who offer prices closer to production costs, with reduced cost per unit compared to retailers^9^ ^,^ ^11^ ^,^ ^18^. Associations or consortia of institutions for joint procurement also indicate that medicine prices are sensitive to economies of scale and negotiating power^1^ ^,^ ^5^.

Stocks improve the level of services by deploying resources needed for the production process and encouraging economies of scale in procurement, protecting against price increases and demand uncertainty^17^. Storage and warehouse operating costs should be added to the costs of medicines in stock, proportional to product specificity (cooling requirements) and physical storage area^4^.

Such elements account for the more prevalent logistics costs of medicines transferred by MH for local distribution, such as ethinyl estradiol + levonorgestrel and NPH and regular insulin, and therefore tend to occupy a lot of storage space and/or demand significant human resources for frequent monthly transfers.

Ipratropium bromide spray and carbidopa + levodopa showed proportionally higher dispensation costs compared to the others, with no clear justification observed. This is probably due to the low costs of the other components, increasing dispensation proportionately. Low volume purchases, extremely specific use instructions, and relatively low consumption demand support this hypothesis.

The differences identified in the comparisons were more favorable to costs at municipal level: SMS-RJ cost estimates resulted in prices below those of ATFP for 20 medicines. The RP paid by MH to private pharmacies is more than four times the price paid by SMS-RJ, considering procurement, logistics and dispensation costs, in eight products. These differences remain relevant even in the best payment scenario (RP minus taxes and copayment by users, when applicable).

The more advantageous results of SMS persist when using the median rather than the average of the percentage differences. The RP for medicines featured in the ATFP list are established considering the factory price approved by *Câmara de Regulação do Mercado de Medicamentos* (Medicine Market Regulation Chamber), information on market revenue and retail trade volume of the medicines, and the average discount on the factory price of the respective medicines^15^. The observed differences certainly raise questions about the possible overestimation of the RP of some pharmaceutical inputs, even though they are determined based on the lowest factory prices approved.

The identified differences intensify the debate raised by the 2011 TCU report. Aggregating the other cost components produced important variations. The percentage difference of 2,507.0% between the median of the municipal cost and the RP of ATFP for the medicine captopril in the abovementioned document fell to 1,325.6% in the most favorable scenario for MH. A similar reduction trend occurred with enalapril: from 1,937.0% to 1,325.6%.

Despite the comparative reductions, the RP paid by MH are still 14 times higher than prices paid by SMS. These angiotensin-converting enzyme inhibitors, recommended for treating systemic high blood pressure and congestive heart failure^10^, are widely used in SUS^6^.

A similar investigation carried out by Carraro^j^ compared the maximum RP paid for 13 medicines available from ATFP, minus taxes, with their costs per PU estimated for 12 municipal health departments in nine states, including logistics and dispensation costs. The RP of ATFP was, on average, 150.1% higher than the estimated costs of municipal pharmacies, and no medicine price paid by MH was inferior to municipal costs.

Although the percentage differences are systematically favorable to estimated municipal costs per PU in both studies, there was a greater disparity in SMS-RJ (3.5 to 28.6 times lower). City size, volume purchases, epidemiological profile and local health system structure, including pharmaceutical services, may have contributed to these findings.

Simulations based on medicine consumption in SMS-RJ in 2012 and expenditures estimated by the lowest RP of ATFP also showed savings in municipal provision, with the difference in overall costs exceeding R$95 million if prices of the ministry program had been used. Items with a RP lower than the estimated municipal cost have low consumption and, analyzed in an integrated way, have a small impact and do not reverse the municipal advantage. The savings correspond to more than three times the amount of federal transfers (R$31,562,221.00) from CBAF to SMS-RJ.

There are other debates related the concurrence of the two pharmaceutical services provision models: the size and overlapping of lists of medicines available from SUS units and ATFP accredited retail pharmacies, which lead to service duplication; lack of use of public sector purchasing power; concentration of the Brazilian Ministry of Health as the key player in supplying medicines for basic care, which is primarily provided at municipal level^12^ ^,^ ^13^.

The advantages of municipal provision found in this study cannot, however, be generalized to other local arrangements and realities. The estimated costs concern a single location. The city of Rio de Janeiro and SMS-RJ have singularities that are not necessarily nor frequently reproduced in Brazilian areas where approximately 80.0% of Brazilian municipalities have up to 30,000 inhabitants. The size of the population and the health service network favor the volume of purchases and increase the city’s power of negotiation. Coupled with the relatively well-structured management of pharmaceutical services, they can potentially reduce costs and maximize efficiency. Finally, other unobserved factors such as the complex logistics in a continental-size country and the different sales taxation schemes of states and municipalities also have repercussions.

The study was limited to a single year, providing a static picture in time. It may not have picked up some relevant cost elements of the municipal pharmaceutical chain that might have emerged in a longer study period, such as seasonality, prescription profile changes, etc.

Administrative costs directly involved in the procurement process and those from loss and misplacement were not included. The former are difficult to be individualized in the pharmaceutical services management chain, especially because the human resources involved in the administrative process of medicine procurement also engage in other procurement activities. This prevents time estimates or the definition of an apportionment criterion capable of identifying this element. Moreover, in general there is only one bidding process per year. The literature on losses is scarce and national statistics are unavailable. Medicine procurement in SMS-RJ is by electronic trading with price registration, with on-demand delivery based on consumption, enabling smaller stocks and minimizing losses from product expiry. Misplacements are covered by insurance provided in the logistics contract, which was included in the specific component costs. The health units have a technical accountability structure and dispensation rules that allow control and reduce misplacement.

This study reinforces the importance of costs in analyzing SUS policies, in particular those related to pharmaceutical services and the provision of medicines by the government. The pharmaceutical services model of the ATFP Program is based on the logic of medicine consumption as a promoter of access, with no emphasis on matters related to costs compared to large public medicine purchasers, who must consider their financial sustainability. The difference in costs between the public provision of SMS-RJ and ATFP indicates that some of the reference prices could be reduced, when compared to prices paid by SMS, which are systematically more favorable.

## Footnotes

a Tribunal de Contas da União. Relatório de auditoria operacional: farmácia popular. Brasília (DF): Tribunal de Contas da União; 2011 [cited 20 Oct 2013]. Available from: http://portal.tcu.gov.br/lumis/portal/file/fileDownload.jsp?inline=1&fileId=8A8182A14D6E85DD014D7327A82819E4

b Câmara dos Deputados. Relatório da CPI: medicamentos. Brasília (DF): Câmara dos Deputados; 2000 [cited 10 Jan 2014]. Available from: http://www2.camara.leg.br/atividade-legislativa/comissoes/comissoes-temporarias/parlamentar-de-inquerito/51-legislatura/cpimedic/relatfinal.html

c Ministério da Saúde. DATASUS. Cadastro Nacional dos Estabelecimentos de Saúde - CNES. Brasília (DF): Ministério da Saúde; 2013 [cited 3 Feb 2013]. Available from: http://cnes.datasus.gov.br/Lista_Es_Municipio.asp?VEstado=33&VCodMunicipio=330455&NomeEstado=RIO%20DE%20JANEIRO

d Ministério da Saúde. Portaria nº 1.555, de 30 de julho de 2013. Dispõe sobre as normas de financiamento e de execução do Componente Básico da Assistência Farmacêutica no âmbito do Sistema Único de Saúde (SUS). *Diario Oficial Uniao*. 3 jul 2013.

e Ministério da Saúde. Portaria nº 971, de 15 de maio de 2012. Dispõe sobre o Programa Farmácia Popular do Brasil. *Diario Oficial Uniao*. 17 maio 2012;seção 1.

f Ministério da Fazenda, Secretaria de Receita Federal. Instrução Normativa SRF nº 162, de 31 de dezembro de 1998. Fixa prazo de vida útil e taxa de depreciação dos bens que relaciona. Brasília (DF): Ministério da Fazenda; 1998 [cited 9 Oct 2014]. Available from: https://www.receita.fazenda.gov.br/Legislacao/ins/Ant2001/1998/in16298.htm

g Ministério da Fazenda. Secretaria de Receita Federal. Instrução Normativa SRF nº 130, de 10 de novembro de 1999. Altera o Anexo I da Instrução Normativa nº 162, de 31 de dezembro de 1998. Brasília (DF): Ministério da Fazenda; 1998 [cited 2014 Oct 9]. Available from: https://www.receita.fazenda.gov.br/Legislacao/ins/Ant2001/1999/in13099.htm

h Carraro WBWH, Mengue SS. Mensuração dos custos totais do fornecimento de medicamentos pela rede básica no Brasil. In: 13º Congresso Internacional de Custos; 18-19 abr 2013 [cited 2014 Jan 10]; Porto, Portugal. Available from: http://www.otoc.pt/news/PENCUSTOS/pdf/076.pdf

i Silva RM. Programa “Aqui tem Farmácia Popular”: expansão entre 2006-2012 e comparação com os custos da assistência farmacêutica na Secretaria Municipal de Saúde do Rio de Janeiro [these]. Rio de Janeiro (RJ):Universidade do Estado do Rio deJaneiro;2014.

j Carraro WBWH. Desenvolvimento econômico do Brasil e o Programa Aqui Tem Farmácia Popular: limitantes e potencialidades [these]. Porto Alegre (RS): Universidade Federal do Rio Grande do Sul; 2014.
